# Incorporation of rhodamine B into male tobacco budworm moths Heliothis virescens to use as a marker for mating studies

**DOI:** 10.1673/1536-2442(2006)6[1:IORBIM]2.0.CO;2

**Published:** 2006-05-05

**Authors:** Carlos A. Blanco, Omaththage Perera, Jeffery D. Ray, Earl Taliercio, Livy Williams

**Affiliations:** 1 USDA-ARS, Southern Insect Management Research Unit, Stoneville, MS 38776; 2 USDA-ARS, Crop Genetics and Production Research Unit, Stoneville, MS 38776; 3 Order of authors determined by alphabetical order of surname; 4 Current address: USDA-ARS Exotic and Invasive Weeds Research Unit, 920 Valley Rd., Reno, NV 89512

**Keywords:** fecundity, spermatophore, mortality, tobacco budworm

## Abstract

Rhodamine B, a dye commonly used in a variety of biological studies was incorporated into the bodies of male tobacco budworm moths, Heliothis virescens (Lepidoptera: Noctuidae), by allowing them to feed freely on 0.1% rhodamine dissolved in a 10% sucrose solution. After exposing males for one to three days to this pigment, rhodamine was clearly detectable in >82% of spermatophores extracted from untreated females. The intake of this dye did not affect the life span, the production of eggs or the capacity of moths to copulate when compared with moths fed only a sucrose solution or water. Rhodamine B was easily identifiable externally but was more apparent internally in males after only one day of exposure to the pigment. Even at this short feeding duration, rhodamine was detectable in >50% of males 5 days after feeding stopped. Longer exposure to the dye significantly increased the percentage stained. Detection of rhodamine was slightly enhanced by the use of ultraviolet light. The dye accumulation in internal abdominal organs was a better indicator of the presence of the pigment than external contamination of the moth. The use of the method described in this report can be a tool for the rapid incorporation of a low cost dye in the tobacco budworm for biological, behavioral and genetic studies.

## Introduction

The tobacco budworm (Helioihis virescens Fabricius) is one of the most damaging pests of cotton in North America and is capable of developing resistance to chemical insecticides (Luttrell et al. 1987, Sparks et al. 1993, Hardee et al. 2001, Teran-Vargas et al. 2005). In order to detect changes in this insect's susceptibility to insecticides, field-collected samples are commonly maintained under laboratory conditions and their offspring are tested. The U. S. Department of Agriculture (USDA)-Agricultural Research Service (ARS) in Stoneville, Mississippi receives from the North America Cotton Belt field samples of H. virescens male moths captured in pheromone traps to test the response of F1 and F2 generations to Bacillus thuringiensis (Berliner) proteins (Blanco et al. 2004). This method of collecting tobacco budworm was an easy and logistically feasible way of obtaining a large number of moths. Since 2003, it has become increasingly difficult to collect these moths in pheromone traps from cotton regions (Parajulee et al. 2004, Blanco et al. 2005). Therefore, it is very important to improve methods to achieve the best genetic representation of these field-collected males.

Development of optimal mating conditions can ensure the greatest genetic representation and this can be assessed by evaluating the copulation frequency of moths. Dissecting a female moth's bursa copulatrix after it has been exposed to males for a fixed period of time is a way of obtaining copulation frequency (Henneberry and Clayton 1985). However, due to the polyandrous habits of H. virescens (LaMunyon 2001), a female bursa copulatrix can contain sperm bundles (spermatophores) from more than one male. It has been observed that laboratory-reared females mass mated with field-collected males acquire an average of 0.68 spermatophores per night (Blanco et al. In Press). The genetic contribution of each of these males under our mass mating conditions can be partially assessed by either including a known proportion of marked males that, having the same opportunity to copulate as those unmarked males, can potentially produce the same proportion of marked spermatophores or by alternative detailed genetic studies.

Different techniques have been used to mark H. virescens. For example, in order to study migration habits Ramaswamy *et al.* (1985) utilized external fluorescent markers applied to moths and internal markers (Calco oil red) fed to larvae. Bell (1988) treated plants in the field with the same purpose utilizing Red Calco and finding 27.5% of the moths internally marked. Stadelbacher (1991) obtained a higher proportion of stained moths (30–86%) with either rubidium or strontium chloride feeding larvae on markers. Hendricks and Graham (1970) fed larvae with a range of pigments obtaining marked spermathophores, however this approach was not useful for marking these sexual structures by feeding moths.

The main objective of this study was to test the potential of rhodamine B for marking male H. virescens moths during free feeding. Is was also of interest to find out if there were any adverse effects on the reproductive capacity of the insects and the durability of the dye in moth bodies. Rhodamine B, is a histological stain that has proven useful for marking insects, for example, Jones *et al.* (1972) utilized it as a spray to mark Heliocoverpa zea moths externally, and Sparks and Cheatham (1973) injected and fed rhodamine B into (Manduca sexta). This study reports modifications for easy, self-feeding, quick and reliable incorporation of rhodamine B into H. virescens bodies and spermatophores to facilitate studies on the genetic contribution of male moths under mass-mating conditions.

## Materials and Methods

Male H. virescens moths (< 36-h old) from the USDA- ARS colony at Stoneville, Mississippi were fed one of the following solutions (treatments): 1) water, 2) 10% (v : v) sucrose solution and 3) 0.1% rhodamine B (Sigma Aldrich, 95% dye content) dissolved in a 10% sucrose solution. Moths had free access to a solution though a sponge (8 × 5 × 2 cm holding about 30 ml) rewetted daily placed on top of cheese cloth in the upper part of carton containers (Neptune© half-gallon [1.89 L] waxed-carton [12.5-cm diameter, 15.5-cm height]). Containers with moths were maintained in an incubator set at 27±1 °C, 75 ± 10% RH, and 14/10 h L:D for a fixed number of days.

### Experiment 1

To obtain information on the minimum number of days necessary to mark spermatophores and the potential detrimental biological effect that feeding on rhodamine might have had on mating frequency and fertility, the following experiment was conducted.

Forty male moths were placed in a container and fed sucrose or rhodamine solutions for one, two or three days. After a specified number of feeding-days (1–3) males were transferred to a 3.78 L carton container (Neptune© gallon waxed-carton [16.5-cm diameter]) where they were mass-mated with unfed (<36-h old) females at a 1:1 ♀ /♂ ratio, <30 per sex density depending on male mortality during feeding. Moths were left to copulate for 1 or 2 days in an incubator under the conditions described above. Live females after the copulation period were divided into two groups: a) half of the females were transferred individually into 0.47 L (pint-size) containers (Neptune© waxed-carton [8.5-cm diameter]) with 15 mL of sucrose solution in a 30-mL plastic cup with a Kimwipe© tissue stuffed into the cup to prevent moths from drowning. A piece of cheese cloth covered the container. Eggs were allowed to accumulate for 2 days on the cheese cloth and were then counted. Fertilized eggs (dark eggs to larval hatching) were recorded two days later.

The other half of the surviving females (b) were immediately frozen and dissected to extract and count spermatophores from their bursa copulatrix. These male sexual structures were examined on a fluorescent microscope (Zeiss™ Axioscop 2,) provisioned with an illuminator (Zeiss™ N HBO 103) and a rhodamine filter (absorption 540 nm/emission 625 nm) (see Illustration 1). This filter optimized visualization of rhodamine-treated (positive resolution) compared with untreated (negative resolution) spermatophores that are virtually invisible to this light. Images of positive and negative resolution were electronically captured utilizing the Zeiss AxioVision (version 2.05) computer program. Four replications were setup for each treatment. Data were analyzed by analysis of variance for randomized complete block design, using SAS 9.1 and means were compared using LSD multiple comparison test at P≤ 0.05.

**Illustration 1.**
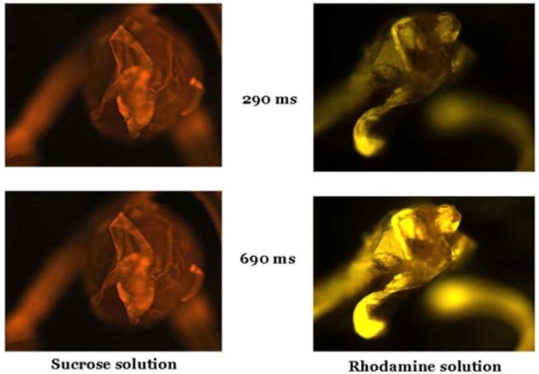
Reflectance of rhodamine B utilizing the same Heliothis virescens spermatophore at two different resolution speeds. Spermatophore on the left (non-rhodamine treated) is used for comparison.

### Experiment 2

A second objective was to assess the potential effect that rhodamine-feeding might have on moth mortality and quantify dye detection in moth bodies. Another experiment was conducted to assess this effect.

A set of six carton containers per treatment (water, sucrose or 0.1% rhodamine solution) with 20 same-sex moths each per replication were set-up to feed for one, two, three or four consecutive days. Sponges that supplied treatments were rewetted daily and removed after the fixed number of days (1 - 4). One randomly chosen container per treatment was rated each day for six consecutive evaluation days to assess moth mortality (0 - 5 days shown in Figures 6 and 7). Surviving moths in containers at each evaluation date were frozen for detection analysis. A sub-sample of ≤12 frozen moths was taken at random from each treatment / evaluation date sample. This experiment was repeated four times on different dates.

Rhodamine detection in frozen moths was evaluated by four different methods. The exterior of the moths was visually inspected (Inspection Method 1, “Visual”) for signs of rhodamine (red coloration) without the use of a magnifying glass or microscope. The same moths were externally inspected under UV light (Spectroline™ 240-C at 254 and 365 nm wavelength) (Inspection Method 2, “UV”). After moths were inspected by methods 1 and 2, moth abdomens were crushed by hand onto a white paper towel to obtain internal fluids. Paper towels with abdomen-fluid stains were visually inspected without (Inspection Method 3, “Paper”) and with (Inspection Method 4, “Paper/UV”) the use of UV-light (254 and 365 nm) (Illustration 2).

**Illustration 2.**
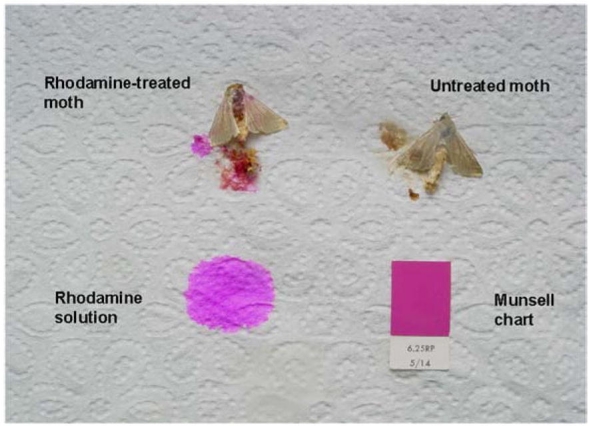
Average number of eggs (A) and percent fertile eggs (B) per Heliothis virescens female after being mated with males fed different solutions (water, sucrose solution or rhodamine B [0.l%] dissolved in 10% sucrose solution) for l to 4 days. NS = not significantly different at P < 0.05. (N = 60).

The experimental design consisted of a randomized complete block. Treatments had a factorial structure with 4 days on rhodamine, sugar solution or water by 6 moth evaluation days. When data were analyzed combined across methods, method was a subunit treatment and the experimental design was a split plot with the main unit design described above. Data were analyzed with Proc Mixed using SAS 9.1 (SAS Institute 2002) and means were compared using LSD test at P≤ 0.05.

## Results and Discussion

**Figure 1.**
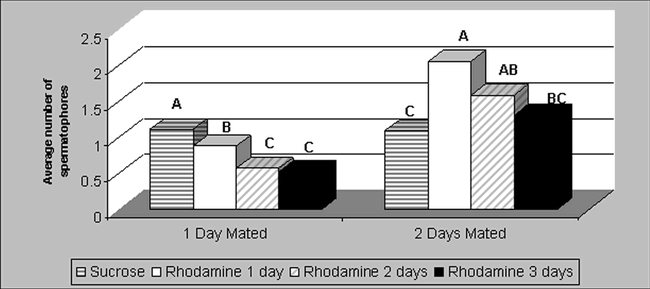
Average number of spermatophores extracted from Heliothis virescens females that were mass mated for 1 or 2 days with males fed sucrose solution or 0.1% rhodamine B for 1 to 3 days. Same letters on bars by days mated are not significantly different at P < 0.05. (N = 60).

**Figure 2.**
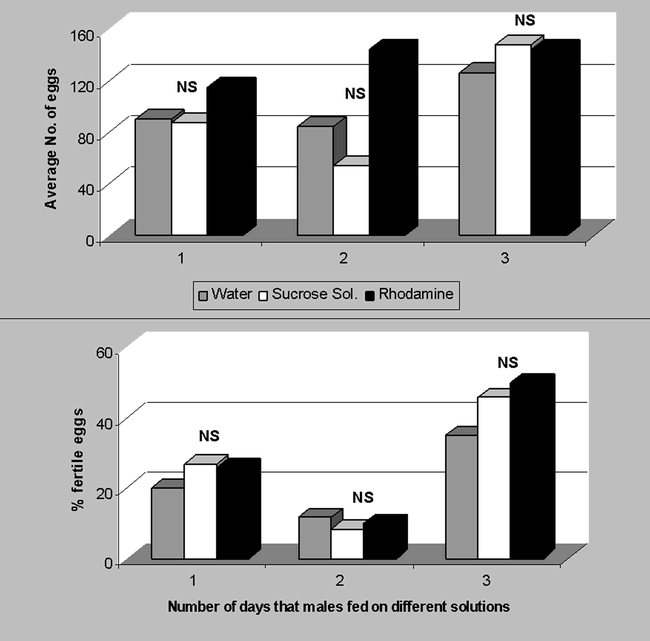
Average number of eggs (A) and percent fertile eggs (B) per Heliothis virescens female after being mated with males fed different solutions (water, sucrose solution or rhodamine B [0.1%] dissolved in 10% sucrose solution) for 1 to 4 days. NS = not significantly different at P < 0.05. (N = 60).

Ideally, a marker should not adversely affect the biology of the insect (Hagler and Jackson 2001). Therefore, the potential detrimental effect of rhodamine on moth reproduction was assessed. The mating success of males, as determined by the average number of spermatophores found in the bursa copulatrix of females after a fixed number of mass mating days was significantly lower between males fed sugar solution and those fed rhodamine (P = 0.0001, df = 12, t = 15.3, Figure 1) when moths copulated for only one day. But the opposite was found (P = 0.006, df = 9, t = 5.23, Figure 1) when moths were allowed to copulate for two days. Since the two mating scenarios did not offer a clear trend, the difference might be due to experimental error and not to affected behavioral aspects. The other potential detrimental aspect of moth reproduction investigated was the average number and percent hatching of eggs. Egg production over a 2-day period and fertility by females that were mated with rhodamine-fed males for one to three days was not significantly different between females mated with males fed only sucrose solution (Figure 2). These two parameters, mating success and percent fertile eggs/female, indicate that males were not significantly affected in their reproductive potential and that the viability of their offspring did not decrease with the use of rhodamine B.

**Figure 3.**
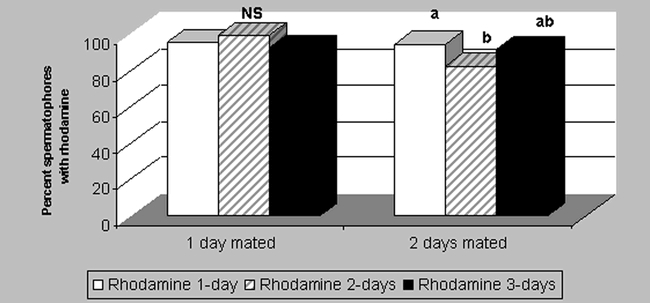
Fluorescent microscopy detection of rhodamine B in spermatophores dissected from Heliothis virescens females that were mass mated for 1 or 2 days with males fed 1 to 3 days 0.1% rhodamine solution. Same letters on bars by number of days mated are not significantly different at P < 0.05. NS = Not significantly different. (N =120).

**Figure 4.**
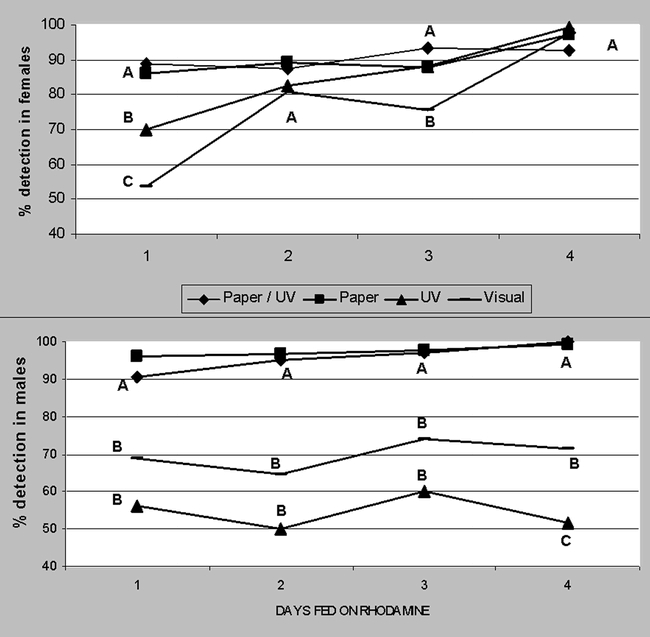
Percent rhodamine B detection in Heliothis virescens moths that were fed this solution for 1 to 4 days. Four different detection methods were employed described in upper graph legend box. Same letters by treatment day by sex are not significantly different at P < 0.05. (N = 48).

**Figure 5.**
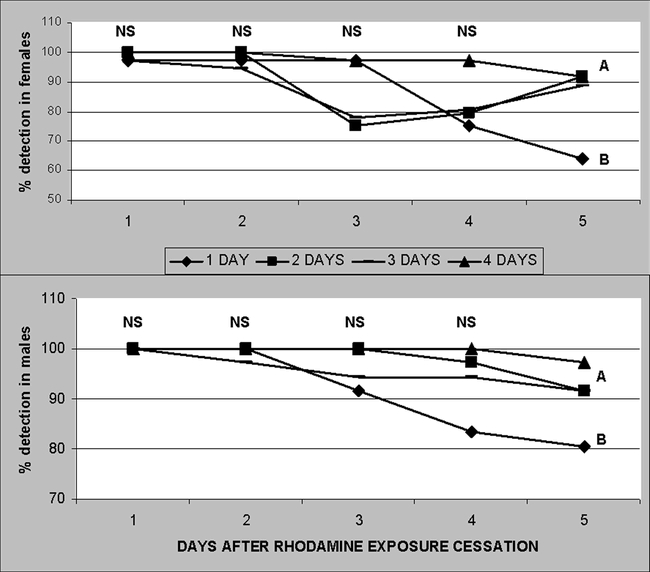
Percent rhodamine B detection in Heliothis virescens moth's abdominal contents after feeding for 1D4 days on 0.1% rhodamine B solution stopped. Similar letters by treatment day by sex are not significantly different at P < 0.05. NS = Not significantly different. (N = 48).

The percentages of rhodamine-stained spermatophores (≥82%, Figure 3) and males that showed rhodamine accumulated in their abdomen (≥95%, Figure 4) aid us in understanding the difference between the number of moths drinking the marker and its detection in sexual bundles. During fluorescent-light inspection of spermatophores from male moths that fed on rhodamine, spermatophores could be clearly distinguished (positive resolution) from those non-stained (negative resolution) (Illustration 1 (#I1)). The positive rhodamine resolution (≥82%) obtained from spermatophores dissected from the female bursa copulatrix indicates that this is a rapid and efficient way of marking H. virescens males. There were no significant differences in the percent detection of marked spermatophores between males fed for one, two or three days and mass mated for only one day. However, significantly lower detection (82%) of spermatophores occurred from males fed two days and mass mated two days (P> 0.0001, df = 9, t = 28.2) than males fed for the same amount of time and mass mated only one day (Figure 5), a fact that we attributed to experimental error. We did not detect any fluorescence in spermatophores of males fed on sucrose solution. These results indicate that incorporation of this marker into spermatophores is achieved rapidly (after only one day) with a success range of 82–100%.

**Figure 6.**
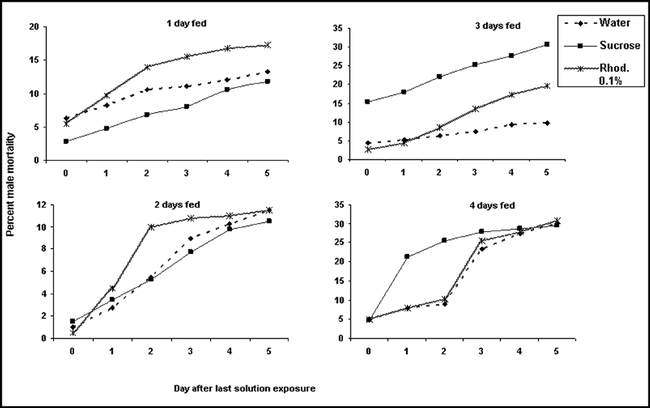
Cumulative Heliothis virescens male mortality after feeding on water, 10% sucrose solution or 0.1% rhodamine B solution for 1–4 days. (N = 48).

**Figure 7.**
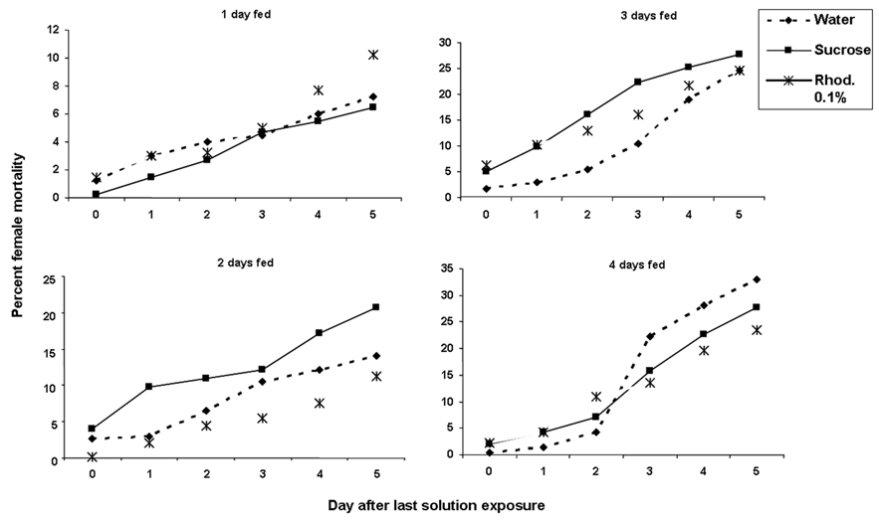
Cumulative Heliothis virescens female mortality after feeding on water, 10% sucrose solution or 0.1% rhodamine B solution for 1–4 days. (N = 48).

In order to determine the proportion of 'false negatives' produced by this technique the percentage of moths that did not ingest the marker nor pass it on to spermatophores was evaluated. Simpler, non-destructive rhodamine-detection methods such as observing moths externally (Detection method 1, referred to as “visual” in Figure 4) without the aid of magnification showed more accumulated dye near the anus and to a lesser extent on the exterior of the mouth parts. At least half of the moths showed visible dye signs 5 days after exposure to rhodamine ceased (Figure 5). This visual technique yielded significantly lower resolution when averaged over all treatments in females (76.9%), and males (69.7%) as compared with crushing the moths on paper towels (Figure 4). Another detection method involved the same visually-inspected moth samples but now inspected under ultraviolet light (Detection method 2, referred to as “UV” in Figure 4) which obtained significantly higher resolution in females and a significantly lower resolution in males (♂P = 0.002, t = 13.2, df = 2. ♀P = 0.006, t = 4.84, df = 4) than visual inspection. Although these two techniques can be used with live moths, they produced significantly lower resolution percentages than the inspection of abdominal contents.

Crushing moth abdomens on a white paper towels (Detection method 3, referred to as “Paper/UV” in Figure 4) shows greater contrast between a rhodamine and sucrose solution fed moth (Illustration 2). Although this high detection (≥90% detection in males, ≥88% in females) but destructive method made rhodamine stains significantly more identifiable in both sexes than external inspection (♀P =0.0005, t = 9.36, df = 4. ♂P =0.002, t =14.1, df = 2). We noticed that handling moths by hand, and not with tweezers, made them defecate, and rhodamine was present in these excretions.

Evaluating moths crushed on paper towels (method 3) under ultraviolet light (Detection method 4, referred to as “Paper/UV” in Figure 4) did not improve pigment detection. However, it did facilitate differentiation between rhodamine and red coloration produced by the bacteria Serratia sp., a common pathogen of H. virescens (Sikorowski and Lawrence 1997). Therefore, the use of ultraviolet light to confirm the presence of dye is recommended due to the fact that this bacterium, unlike rhodamine, does not fluoresce under ultraviolet light. Method 4 was useful for detecting dye ingestion and retention in moths (Figure 5).

Feeding moths with this dye solution resulted in similar marking percentages as those obtained by Sparks and Chetham (1973) with the injection of rhodamine directly into tobacco hornworm moths. However, unlike these authors reported, we did not detect rhodamine in tobacco budworm spermatophores with the use of UV light.

Rhodamine B did not significantly reduce the life span of either sex in tobacco budworm (♀P = 0.57, 0.41, 0.11, and 0.57; ♂P = 0.31, 0.19, 0.40, and 0.38, when fed for one to four days respectively, Figures 6 and 7). There was only a significant difference in mortality between females fed three days sucrose solution and water (P = 0.01 t = 3.2, df = 6, Figure 7). The trend towards higher mortality as feeding days increased with the three treatments might be attributed to natural moth mortality and not to any adverse effect of the dye, suggesting that this dye does not reduce the lifespan of either sex in this moth. Because rhodamine can be detected in spermatophores and insect bodies after only one day of exposure, this might reduce unnecessary rhodamine-feeding time, thus allowing more days to perform desired experiments.

Cost and ease of use are important attributes for a marker (Hagler and Jackson 2001). The cost of this dye for producing a liter of rhodamine-treated sucrose solution at the tested concentration was $0.35 (2005 prices), and one liter of this solution was sufficient for marking ≥2,000 moths. Additionally, this methodology is easy to implement and does not require any special skill such as injecting the dye into the moth's body (Sparks and Cheatham 1973) or special spraying equipment to mark the moths externally (Jones et al. 1972). However, for a very small moth sample this procedure is less efficient and it would take longer (≥ 1 day) as compared with injecting or spraying a marker, although no shorter rhodamine exposure (i.e. < 24-h) was investigated to overcome this minor disadvantage. However, feeding rhodamine can be extremely useful for marking hundreds of moths at once. This technique allows the rapid incorporation of a dye without intensive work and/or numerous and skilled research personnel, it has a relatively low cost and can be applied to any number of moths. It also overcomes the potential effect that long-term manipulation of field-collected insects (i.e. rearing larvae in artificial diet) under laboratory condition might have in reproduction and/or behavior of moths. One to three days of feeding rhodamine to H. virescens, while the moths mature sexually, for example, is all that is required to obtain a reliable spermatophore marker.
